# Health literacy and use of preventive health services among North Korean defectors in the Republic of Korea

**DOI:** 10.1371/journal.pone.0195964

**Published:** 2018-06-27

**Authors:** In Gyu Song, Haewon Lee, Jinseon Yi, Min Sun Kim, Ichiro Kawachi, Sang Min Park

**Affiliations:** 1 Central Hospice Center, National Cancer Center, Goyang-si, Gyeonggi-do, Republic of Korea; 2 Department of Family Medicine, Seoul Medical Center, Seoul, Republic of Korea; 3 Department of Community Health Nursing & Nursing Care System, Seoul National University College of Nursing, Seoul, Republic of Korea; 4 Department of Pediatrics, Seoul National University Hospital, Seoul, Republic of Korea; 5 Department of Social and Behavioral Science, Harvard T.H. Chan School of Public Health, Boston, MA, United States of America; 6 Department of Family Medicine & Biomedical Sciences, Seoul National University College of Medicine, Seoul, Republic of Korea; 7 Department of Global and Population Health, Harvard T.H. Chan School of Public Health, Boston, MA, United States of America; Boston University, UNITED STATES

## Abstract

It is known that some groups of immigrants can have low health literacy and it may affect their health. Although the number of North Korean defectors in the Republic of Korea has increased, little is known about their health literacy and health behavior. Adult North Korean defectors (n = 399) in this study were surveyed on health literacy, using the Korean Health Literacy Scale, and on the use of preventive services. Those with better health literacy scores were more likely to have vaccination than the lower scoring group (adjusted OR = 2.44; 95% CI, 1.19–5.00). However, undergoing medical check-up may not be associated with health literacy. In subgroup analysis, among defectors who lived alone (*P* = 0.032) or who had longer time in other countries before entering (*P* = 0.007), the vaccination coverage was associated with their health literacy scores. This study provides evidence for an association between health literacy and influenza vaccination coverage among North Korean defectors even though they may have fewer language barriers than other immigrants. Further research is needed to develop interventions for improving health literacy and their preventive health behavior.

## Introduction

From 1995 to 2000, millions of people died from starvation and hunger-related diseases in North Korea. This period is called the “March of Hardship” [1]. Since the late 1990s, many North Koreans escaped to the Republic of Korea (Korea) through other Asian countries, for political or economic reasons. Until recently, about 150 defectors entered every month; roughly 30,000 defectors had settled in Korea by August 2016, accounting for about 0.06% of the Korean population [2]. About 70% of defectors are women and those aged less than 30 years comprise 45% of them. Half of the defectors did not have jobs when they lived in North Korea and about 80% of defectors had received education up to the high school levels or lower. About a quarter of them were recipients of basic livelihood grants and the economic activity participation rate was 57.9% [3]. The defectors had many health problems, such as malnutrition, infections, and trauma from when they lived in North Korea and other countries. These problems affected their health even after entry, and made their settlement in Korea difficult [4, 5]. They are given health examinations immediately after entering Korea, and confirmed diseases are treated. After verification of their identity, the Korean government provides financial and medical support under the Medical Care Assistance Act for up to the first 5 years. Notwithstanding this support, North Korean defectors still have difficulties adjusting to the Korean healthcare system and practicing self-care [6]. The health finance system of Korea achieved universal population health coverage based on social health insurance (97%) and medical aid (3%). However, benefit coverage is limited (about 60%) and out-of-pocket payment still accounts for a significant portion, which lays a burden on vulnerable populations [7]. North Korean defectors receive medical assistance for the first 5 years in Korea, and over half will remain on assistance after this period because of their economic status [8]. The government support for some preventive services such as vaccination for the elderly and children or regular medical check-up for population in criteria [9].

As medical care has become increasingly sophisticated, the importance of health literacy is greater than ever [10]. Dimension of health literacy is beyond function in the role of patient, such as a citizen in the political arena or as a member of the audience in relation to the media [11, 12]. According to Sørensen et al., “Health literacy is linked to literacy and entails people’s knowledge, motivation and competences to access, understand, appraise, and apply health information in order to make judgments and take decisions in everyday life concerning healthcare, disease prevention and health promotion to maintain or improve quality of life during the life course” [12]. Nonetheless, some groups, such as immigrants, the elderly, and those with limited formal education and low income, are more likely to be less literate [10, 13]. Because of language and cultural barrier, immigrants can have inadequate level of health literacy, especially in the less educated group [14, 15, 16]. Previous studies suggested that people with inadequate health literacy were less likely to report they had received preventive health care [16, 17]. Patients with low health literacy have poor health status and higher hospitalization rates, which increases healthcare system costs [12]. People with low health literacy may have difficulty understanding a doctor’s instructions and recommendations, or health education and promotion materials, and thus may not have adequate information on influenza vaccination [14]. Previous studies show possible pathways between patient health literacy, participation in the health-care process, and population health [18]. Distinct from other immigrants or refugee groups, North Korean defectors speak the same language as the indigenous people. Yet, cultural barriers and economic challenges still hinder access to, and understanding of, relevant health information [6]. However, studies on health literacy of the defectors are, to the best of our knowledge, absent from the literature.

We assumed that North Korean defectors with low health literacy may be less likely to use preventive services, and it could contribute to the poor health status. However, little is known about the defectors’ health literacy status. The aim of our study was to determine the association between health literacy and use of preventive healthcare services, such as self-reported influenza vaccination coverage and medical check-up rates, among North Korean defectors.

## Materials and methods

### Ethical considerations

The survey protocol was granted an exemption by the Institutional Review Board at the Seoul National University Hospital, because this was a secondary analysis of de-identified data (IRB No. 1503-019-654).

### Study design and data source

This was a cross-sectional study to assess the relationship between North Korean defectors’ health literacy and their preventive health behavior. The data gathered were part of a survey performed by the North Korean Refugees Foundation, to research the actual condition of North Korean defectors who had settled in Korea. We were offered this data after personal information was removed. The survey was conducted from August to December 2012. From about 24,000 North Korean defectors living in Korea, 430 were selected by the snowball sampling technique, with consideration of the gender and age distribution of the defectors’ population. In this manner, North Korean defector recruiters introduced some participants, and the participants subsequently referred their acquaintances to the researchers. The surveyors comprised 4 North Korean defectors who were trained for the survey; they met each subject, explained the purpose of the study, and received a consent form from each survey. The participants answered the questionnaire personally, with assistance from the surveyors. Among 430 defectors, 31 respondents aged under 20 were excluded, and 399 participants aged 20 and over were identified.

The survey consisted of questions to determine demographics (gender, age, marital status), variables related to escape or emigration (duration of stay, both in other countries before entry, and in Korea), use of preventive health services, and health literacy. The clinical preventive services that we examined were self-reported influenza vaccination during the prior year, and medical check-ups within 2 years. In Korea, the government recommends medical check-ups every 2 years for people over 40 years of age and for those with national health insurance. Laborers or those on medical assistance under 40 years-old can receive the same benefit. It consists of physical examination and consultation by physician, chest x-ray, some laboratory tests [9]. In cases where the individuals were unable to remember their vaccination and medical check-up history, we treated the information as missing values. To measure health literacy, we used a shortened form of the Korean Health Literacy Scale (S-KHLS). The KHLS was developed for the elderly in 2009. It contains 24 items, and assesses reading comprehension, numeracy, and health-related terms in a variety of Korean healthcare contexts, such as health education materials, newspaper articles, and self-care practices in everyday life [19]. To reduce respondent fatigue, the KHLS was shortened to 12 items, and the internal consistency was confirmed [20]. Five questions asked about health-related terms, and 7 were related to comprehension and numeracy [20]. Because the criterion score for limited health literacy has not been developed for either the KHLS or the S-KHLS, we divided the score into 3 categories, with respect to the distribution of the respondents and mean score in the previous and present studies [21]. The lowest group scored 0 to 9, the middle group scored 10 to 11, and the highest group scored 12. We also performed subgroup analysis for each dimension of S-KHLS using the following categories: health-related terms (0–3, 4, 5), and comprehension and numeracy (0–5, 6, 7).

### Statistical analysis

Data analysis was performed using STATA 12.1 (StataCorp, College Station, TX, US) and RStudio Version 1.0.153. Multitrait scaling analysis was used to examine item convergent validity. Item convergent validity would be acceptable if the correlation between an item and its dimension was ≥0.4. Rasch analysis was also conducted to examine the scale and the data. Statistical differences in vaccination coverage and the percentage who had received medical check-up by health literacy groups were assessed by chi-square tests. Univariable logistic regression analysis was performed to estimate the odds ratio (OR) and 95% confidence interval (CI) of preventive health behavior according to the health literacy score, using the lowest scoring group as reference. Multivariable logistic regression was conducted, and ORs were calculated, following adjustment for age, gender, and marital status in model 1. In model 2, variables related to escape and migration (duration of stay, both in other countries before entry, and in Korea) were used for adjustment, in addition to model 1. We divided each demographic group into 2, based on the median heath literacy score, and calculated adjusted proportions to analyze which demographic group was easily affected by health literacy when accessing preventive health care. P-values less than 0.05 were considered significant, and 95% CI values were calculated to show the strength of the association.

## Results

Table 1 presents sociodemographic and preventive health behavior for the 399 people in the sample. More than 80% of the respondents were aged under 50, and 73.3% were female. Over 80% of the respondents lived in other Asian countries for more than 1 year, and have lived in Korea more than 4 years. The influenza vaccination coverage in the prior year was 31.1%, and about 60% of the defectors underwent medical check-up within 2 years. In Rasch analysis, infit indices were within 0.8–1.2 and the item reliability was 0.61 (Table in the S1 File). Cronbach’s alpha coefficient of S-KHLS in this study was 0.77. The multitrait scaling analysis showed that dimension correlation coefficients of all items were above 0.30 but less than 0.40. The median overall score on the S-KHLS was 11.

**Table 1:** Demographics and preventive health characteristics of North Korean defectors in the Republic of Korea.

Demographic characteristic	North Korean defectors, n (%) (N = 399)
**Age, years, mean ± SD**	41.0 ±10.0
**20–29**	47 (11.8)
**30–39**	134 (33.6)
**40–49**	142 (35.6)
**50–59**	53 (13.3)
**≥ 60**	23 (5.8)
**Gender**	
**Men**	106 (26.5)
**Women**	293 (73.3)
**Marital status**	
**Married or Living together**	202 (50.9)
**Single**	195 (49.1)
**Duration of stay abroad before entrance (years)**	
**< 1**	66 (17.0)
**1–3**	141 (36.3)
**4–6**	119 (30.7)
**≥ 7**	62 (16.)
**Duration of stay in the Republic of Korea (years)**	
**≤ 3**	69 (17.3)
**4–6**	153 (38.3)
**7–9**	127 (31.8)
**≥ 10**	50 (12.5)
**Influenza vaccination coverage**	
**Yes**	116 (31.1)
**No**	257 (68.9)
**Medical check-up (within 2 years)**	
**Yes**	193 (58.5)
**No**	8 (41.5)

After adjustment for sociodemographic factors, those with better health literacy scores (S-KHLS = 12) were more likely to have been vaccinated during the prior year than the lower scoring group (S-KHLS ≤ 9) on multivariate analysis (adjusted odds ratio [aOR] = 2.14; 95% CI, 1.04–4.41). Further adjustment for escape- and migration-related factors attenuated the association, but this trend remained, and the multivariable OR for the highest versus the lowest scoring group was 2.10 (95% CI, 1.02–4.35) (Table 2).

**Table 2:** Multivariate analysis for influenza vaccination coverage among North Korean defectors according to health literacy score.

Total score (%)	0–9 (19.1)	10–11 (44.1)	12 (36.8)	*P* for trend
No. of subjects^†^ (%)	13 (19.7)	58 (35.2)	45 (31.7)	0.071
Crude	1.00	2.21 (1.11–4.39) ^*^	1.89 (0.94–3.82)	0.194
Model 1^‡^	1.00	2.44 (1.20–4.96) ^*^	2.14 (1.04–4.41) ^*^	0.104
Model 2^§^	1.00	2.44 (1.19–5.00) ^*^	2.10 (1.02–4.35) ^*^	0.119
Health related terms score (%)	0–3 (12.3)	4 (26.1)	5 (61.7)	*P* for trend
No. of subjects^†^ (%)	6 (14.3)	30 (31.6)	80 (33.9)	0.040
Crude	1.00	2.77 (1.05–7.28) ^*^	3.08 (1.24–7.61) ^*^	0.028
Model 1^‡^	1.00	3.13 (1.68–8.40) ^*^	3.69 (1.46–9.38) ^*^	0.011
Model 2^§^	1.00	3.10 (1.14–8.42) ^*^	3.60 (1.41–9.20) ^*^	0.014
Comprehension & Numeracy score (%)	0–5 (22.8)	6 (28.1)	7 (49.1)	*P* for trend
No. of subjects^†^ (%)	15 (18.3)	44 (42.7)	57 (30.3)	0.002
Crude	1.00	3.33 (1.68–6.59) ^*^	1.94 (1.02–3.69) ^*^	0.208
Model 1^‡^	1.00	3.64 (1.80–7.36) ^*^	2.13 (1.11–4.12) ^*^	0.117
Model 2^§^	1.00	3.47 (1.70–7.09) ^*^	2.10 (1.08–4.09) ^*^	0.130

^*^ *P* < 0.05

^†^ Number of subjects who got influenza vaccination during the prior year.

^‡^ Adjusted for age, gender, marital status.

^§^ Adjusted for duration of stay in other countries before entry, duration of stay in the Republic of Korea in addition to model 1.

There was no significant correlation between the results of the test and undergoing medical check-up in this survey group. The proportion of people who underwent a medical check-up within 2 years was highest in the middle scoring group and lowest in the highest scoring group (Table 3). The results did not differ when subjects over 40 years-old were analyzed separately using sensitivity analysis (not shown).

**Table 3:** Multivariate analysis for medical check-ups among North Korean defectors according to health literacy score.

Total score (%)	0–9 (19.1)	10–11 (44.1)	12 (36.8)	*P* for trend
No. of subjects^†^ (%)	38 (59.4)	80 (63.0)	75 (54.0)	0.323
Crude	1.00	1.16 (0.63–2.15)	0.80 (0.44–1.46)	0.308
Model 1^‡^	1.00	1.59 (0.82–3.08)	0.98 (0.52–1.86)	0.614
Model 2^§^	1.00	1.42 (0.72–2.82)	0.90 (0.47–1.73)	0.481
Health related terms score (%)	0–3 (12.3)	4 (26.1)	5 (61.7)	*P* for trend
No. of subjects^†^ (%)	28 (73.7)	43 (62.3)	122 (54.7)	0.069
Crude	1.00	0.59 (0.25–1.41)	0.43 (0.20–0.93) ^*^	0.023
Model 1^‡^	1.00	0.82 (0.33–2.07)	0.53 (0.23–1.18)	0.050
Model 2^§^	1.00	0.73 (0.27–1.95)	0.46 (0.19–1.07)	0.029
Comprehension & Numeracy score (%)	0–5 (22.8)	6 (28.1)	7 (49.1)	*P* for trend
No. of subjects^†^ (%)	43 (54.4)	52 (65.0)	98 (57.3)	0.362
Crude	1.00	1.55 (0.82–2.94)	1.12 (0.66–1.92)	0.871
Model 1^‡^	1.00	1.91 (0.97–3.75)	1.32 (0.75–2.34)	0.508
Model 2^§^	1.00	1.87 (0.93–3.76)	1.24 (0.69–2.24)	0.672

* *P* < 0.05

^† ^Number of subjects who had medical check-up within 2 years.

^‡^ Adjusted for age, gender, marital status.

^§^ Adjusted for duration of stay in other countries before entry, duration of stay in the Republic of Korea in addition to model 1.

When we performed subgroup analysis by each dimension of the S-KHLS, the score for the health-related term dimension was significantly associated with influenza vaccination coverage and the trends in the OR remained significant (p for trend = 0.014), after adjusting for other factors in model 2 (Table 2). However, the score was inversely related to the rate of medical check-ups, but was not statistically significant after adjustment (Table 3). The score for the comprehension and numeracy dimension was also significantly related to the vaccination coverage (Table 2), but there were no correlations between the score and medical check-up history (Table 3).

**Fig 1:**
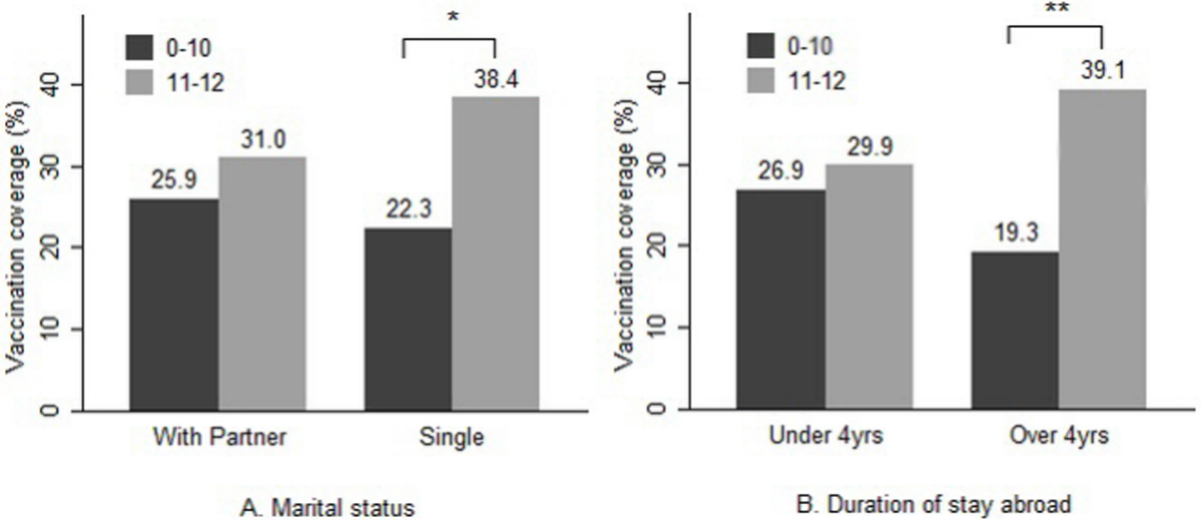
Comparison of influenza vaccination coverage of North Korean defectors by the health literacy score. Influenza vaccination coverage among North Korean defectors, adjusted for respondent characteristics (age, gender, marital status, duration of stay in other countries and Korea). Comparison of vaccination coverage by the score on the health literacy scale. A. When compared to influenza vaccination coverage among defectors living with others, influenza vaccination coverage among defectors living alone was affected by the lower health literacy scores. B. Influenza vaccination coverage among defectors who lived in other countries for more than 4 years before entering Korea was affected by the lower health literacy scores, while the vaccination coverage was independent of the health literacy scores among those who had stayed for a shorter period. *P < 0.05, ** P < 0.01.

After adjustment for sociodemographic factors (gender, age, marital status), and escape- and migration-related factors (duration of stay, both in other countries and in Korea), the vaccination coverage among defectors who lived alone and who had lower health literacy scores was lower than that among those with higher health literacy scores (*P* = 0.032); however, the coverage among defectors with partners was not affected by the health literacy score (Fig 1A). The vaccination coverage among respondents who had lived in other countries for more than 4 years was associated with health literacy scores, while it was not correlated with the health literacy scores among those who had stayed for a shorter period (*P* = 0.007) (Fig 1B).

**Fig 2:**
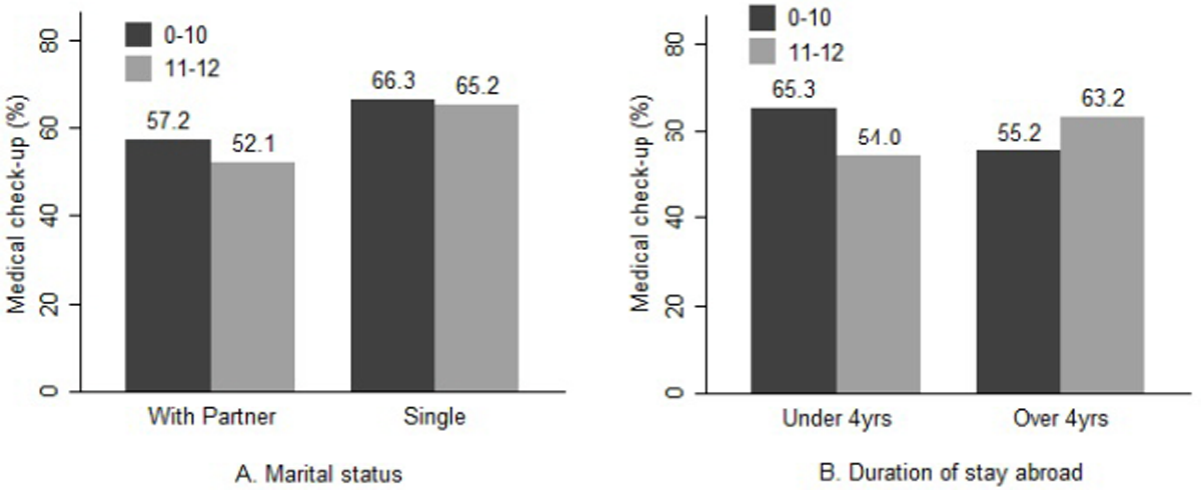
Comparison of regular medical check-up rate among North Korean defectors by the health literacy score. Proportion of medical check-up rates within 2 years among North Korean defectors, adjusted for respondent characteristics (age, gender, marital status, duration of stay in Korea). Comparison of the proportion of individuals who underwent medical check-ups according to the score on the health literacy scale. A. The proportion of those who underwent medical check-up within 2 years was not different according to the health literacy level, even after analysis based on marital status. B. The proportion of those who underwent medical check-up within 2 years was not different according to the health literacy level, when analyzed based on duration of stay in other countries before entry.

However, the proportion of those who underwent medical check-up within 2 years was not different according to the health literacy level, when analyzed according to marital status or duration of stay in other countries before entry (Fig 2).

## Discussion

Our study suggests North Korean defectors with high health literacy might be more likely to have influenza vaccination, while medical check-up within 2 years was not associated with health literacy. To our knowledge, this is the first study to assess the association between health literacy and use of preventive health care among North Korean defectors.

Demographic data from our study showed that the number of women was three times that of men and reflected the North Korean defectors' demographic structure. Because many women who were living in the border region with China are in trade with China, they were easier than men across borders. Some women were trafficked to China and escaped to Korea a few years later. Therefore, North Korean women are dominant in Korea [2].

Several studies reported that people with inadequate health literacy were more likely to report failure to receive influenza vaccination [14, 17]. Our results show a similar trend in total score and each domain (terms score, comprehension and numeracy score), even after controlling for other confounding factors related to escape and migration. Some researchers suggested that interventions to improve health literacy might be useful to reduce disparities in accessing preventive health care [17]. This opinion was also suggested in previous studies on immigrant health and they expected that health literacy can be an important factor to improve the service utilization of immigrants [22–24]. Several specific design features, for example, presenting essential information by itself, or presenting essential information first, and adding video to verbal narratives, can be used to improve comprehension in low health literacy populations. Mixed strategy intervention is also more effective for improving health literacy and accessing healthcare services than a single strategy [25, 26]. Therefore, it is necessary to tailor interventions to improve North Korean defectors’ health literacy, and their knowledge of influenza infection and vaccination. It would also be helpful to educate healthcare providers about health literacy and communication skills [26].

This study shows that the health literacy level of the defectors might not be associated with the use of regular medical check-up. In Korea, the government sends an official document to each individual to arrange for a medical screening and cover the examination fee. Because the defectors receive information about regular free medical check-ups, the health literacy level might not affect use of this benefit. The government recommends influenza vaccination for people over age 50, and provides it free-of-charge to the elderly (over 65) and some underserved classes of people, including North Korean defectors. However, information about public policy for influenza vaccination was not given to each individual. These differences in public health policies might result in differences between results for influenza vaccination and medical check-up in this study. In addition, medical check-up may be affected by the existence of underlying health problems [27]. That is, people seek check-ups not only for prevention, but also because they are feeling sick. This may explain the lack of a monotonic association between health literacy and check-ups.

In our analysis, the vaccination coverage for the defectors who lived alone or who had lived in other countries for over 4 years before entry into Korea appeared to be more affected by health literacy. The social support of the defectors who live alone may be weaker than that of people who have a housemate. Therefore, individual traits, such as health literacy, might affect their health behavior [28]. Future studies will be required to assess the moderating effect of social support or migration-related factors on the association between the defectors’ health literacy and use of preventive services.

Although this study provides some information about the association between health literacy and preventive healthcare use among North Korean defectors, it has significant limitations. First, because we used the snowball sampling technique when we selected survey participants, it was not a representative sample of defectors, and thus it is difficult to generalize the results. However, North Korean defectors are distributed throughout the country, and some do not wish to reveal themselves; thus, non-probabilistic sampling to recruit a large number of defectors was inevitable. This technique has been commonly used in studies of North Korean defectors [29, 30]. Second, this study is based on self-reported information on influenza vaccination and medical check-up, which could be a source of potential error. However, self-reporting of preventive services has been validated as highly sensitive and defines a high level of agreement [31, 32]. Third, we could not access the information on education level which is known for affecting preventive health behaviors or health literacy [10, 33]. North Korean education is known for focusing on ideology and even it does not work normally after economic crisis in 1990th. Even though students are registered in schools, they would go out to earn money or get food instead of studying in school [34]. Thus, we assumed that the association between education level and health literacy may not be as strong as in other populations. Further study including information on education level will be needed. Fourth, Although Cronbach’s alpha coefficient of the S-KHLS in this study was 0.77, this tool was devised for the elderly in Korea, not for North Korean defectors [19]. In our study, the mean of the total health literacy score among North Korean defectors was similar to that among Korean elderly patients with hypertension in the previous study (10.52 vs.10.52) [20], while the mean age of our subjects was younger than that of the study (41.1 vs. 66.6). As older people might have more limited health literacy than younger people, we postulate that North Korean defectors might have worse health literacy compared to Koreans, even though they speak the same language [10]. Further research is needed to develop health literacy assessment tools for North Korean defectors.

## Conclusions

The results of this study provide evidence for an association between health literacy and influenza vaccination coverage among North Korean defectors in Korea even though they may have fewer language problems than other immigrants. However, medical check-ups that the government supports actively, were not associated with health literacy. In order to promote defectors’ health, clinical research and public policy will be required to refine S-KHLS for defectors or develop tools for measuring health literacy; culturally appropriate educational interventions with various resources will be required to improve health literacy and provide defectors with information about health services [13, 14]. As the numbers of immigrants worldwide increased over the last decade, health problems of these populations became a burden in many countries [35]. This perspective on health literacy of defectors is expected to be applied to immigrant health policies of other countries.

## Supporting information

S1 File Rasch-based item characteristics of the short form of the Korean Health Literacy Scale for North Korean defectors. (DOCX)

S2 File Korean version of shortened form of the Korean Health Literacy Scale. (DOCX)

S3 File Minimal data set. (XLS)
