# Supplementary material for: Health literacy and use of preventive health services among North Korean defectors in the Republic of Korea
Source: PLoS One. 2018 Jun 27;13(6):e0195964. doi: 10.1371/journal.pone.0195964 (PMC6021060; doi:10.1371/journal.pone.0195964)
Supplement: S1 File — (DOCX) [file pone.0195964.s001.docx]

| Number | Item Content | Infit | Outfit |
| --- | --- | --- | --- |
| Health-related terms | | | |
| 1 | Alcohol | 1.04 | 2.04 |
| 2 | Obesity | 0.81 | 0.43 |
| 3 | Disease | 1.01 | 1.03 |
| 4 | Hypertension | 0.87 | 0.30 |
| 5 | Lifestyle disease | 1.12 | 1.17 |
| Comprehension and Numeracy | | | |
| 6 | Calculation of intake/output | 0.93 | 1.18 |
| 7 | Next appointment (date) | 1.06 | 1.47 |
| 8 | Clinic schedule (department) | 1.12 | 0.68 |
| 9 | Medication time | 0.95 | 0.97 |
| 10 | Graph of chronic disease statistics | 1.12 | 0.98 |
| 11 | Calculation of liquid medication dose | 0.95 | 0.95 |
| 12 | Nutrition facts | 1.02 | 1.02 |

Table. Rasch-based item characteristics of the short form of the Korean Health Literacy Scale for North Korean defectors (N = 399)
