# Supplementary material for: Health literacy and use of preventive health services among North Korean defectors in the Republic of Korea
Source: PLoS One. 2018 Jun 27;13(6):e0195964. doi: 10.1371/journal.pone.0195964 (PMC6021060; doi:10.1371/journal.pone.0195964)
Supplement: S2 File — (DOCX) [file pone.0195964.s002.docx]

| **Health related term** |
| --- |

※ Please select the appropriate word for each question considering the context of each sentence.

| Example) Old age, pregnant women, patients with high fever and patients with ----- disease should be cautious using spa  ① Electrocardiography ② cardiac (Answer: 2) |
| --- |

| 1. Within two hours after ------, he or she should be cautious using the spa.   ① drinking ② having cancer |
| --- |

| 2. This is about child obesity. Please select the appropriate word.  2-1. ---- means overweight.  ① Anemia ② Obesity  2-2. Obesity can cause various ----  ① disease ② influenza  2-3. Obese children can easily induce -----  ① hypotension ② hypertension  2-4. Obese children can easily induce ----- , such as diabetes which is caused by insulin secretion disability  ① addiction(alcohol) ② health behavior disease |
| --- |

| **Comprehension & Numeracy Part** |
| --- |

※ Please select the appropriate answer.

| 1. To drink total 1,000㎖ of water for a day with a cup(volume: 200㎖), how many cups should you drink?   ① 2 cups ② 5 cups ③ 7 cups ④ 10 cups |
| --- |

※ After reading the < Care description Guide> below, please write down the answer.

| \|  \| \|  \|  \|  \|  \|  \| \| \| --- \| --- \| --- \| --- \| --- \| --- \| --- \| --- \| \|  \| \| \| Care description Guide \| \| \| Cardiovascular Hospital \| \| \|  \| \| \|  \| \|  \| \| \| \| Registered number: 1234567  Attending physician: Dr. Hue Jun \| \| \| \| \| \| \| \| \| Patient name: Gil Dong Hong, Date: September 8^th^, 2007 \| \| \| \| \| \| \| \| \|  \| \| \|  \|  \|  \|  \| \| \|  \| Date of reservation \| \| 10:30 AM, September 8^th^, 2007 \| \| \| \|  \| \|  \| \| \|  \|  \|  \|  \| \| \|  \| Where to go for next visit \| \| Electrocardiography room, 2^nd^ floor ( √ ) \| \| \| \|  \| \|  \| Echocardiography room, 1^st^ floor ( ),  Treadmill Examination room, 1^st^ floor( ) \| \| \| \|  \| \|  \| Administrative Office, 1^st^ floor ( ) \| \| \| \|  \| \| Please visit the room checked √ \| \| \| \| \| \| \| \|   2. When is the next appointment date? YYYY/MM/DD |
| --- | --- | --- | --- | --- | --- | --- | --- | --- | --- | --- | --- | --- | --- | --- | --- | --- | --- | --- | --- | --- | --- | --- | --- | --- | --- | --- | --- | --- | --- | --- | --- | --- | --- | --- | --- | --- | --- | --- | --- | --- | --- | --- | --- | --- | --- | --- | --- | --- | --- | --- | --- | --- | --- | --- | --- | --- | --- | --- | --- | --- | --- | --- | --- | --- | --- | --- | --- | --- | --- | --- | --- | --- | --- | --- | --- | --- | --- | --- | --- | --- | --- | --- | --- | --- | --- | --- | --- | --- | --- | --- | --- | --- |

※ <Treatment Schedule for outpatients> is written below. Please select the appropriate answer.

| < Treatment Schedule for outpatients>   \| Department \| Physician \| Specialty \| Mon \| Tue \| Wed \| Thur \| Fri \| \| --- \| --- \| --- \| --- \| --- \| --- \| --- \| --- \| \| Internal Medicine \| Dr. Sun-Sin Lee \| Gastroenterology \|  \|  \|  \| ● \|  \| \| Dr. Bo-Ram Kim \| Respiratory Disease \| ● \|  \| ● \|  \| ● \| \| General Surgery \| Dr. Hye Hwang \| Upper gastrointestinal, Hepatobiliary \| ● \|  \| ● \|  \| ● \| \| Pediatrics \| Dr. Sun-Hye Lim \| Pediatric Disease \| ● \| ● \| ● \| ● \| ● \| \| Ophthalmology \| Dr. Jin-Ju Oh \| Cataract, Glaucoma \|  \|  \| ● \|  \|  \| \| Dermatology \| Dr. Bo-Bea Jung \| Dermatitis \|  \| ● \|  \| ● \|  \|   3. Which department should you visit if you have symptoms of Cataract?  ① Pediatrics ② Ophthalmology ③ Dermatology ④ General Surgery |
| --- | --- | --- | --- | --- | --- | --- | --- | --- | --- | --- | --- | --- | --- | --- | --- | --- | --- | --- | --- | --- | --- | --- | --- | --- | --- | --- | --- | --- | --- | --- | --- | --- | --- | --- | --- | --- | --- | --- | --- | --- | --- | --- | --- | --- | --- | --- | --- | --- | --- | --- | --- | --- | --- | --- | --- |

※Please select the appropriate answer, according to the drug prescription below.

| \|  \| \| \| \| \| --- \| --- \| --- \| --- \| \| Gil-Dong Hong \| Oncology \| Date: \| 2007-09-08 \| \| Registered Number: 1234567 \| \| Age: 37 \| \|  \| \|  \| \| \| \| \| Take your medicine three times a day every eight hours, one tablet each time. \| \| \| \| \|  \| \| \| \| \| < Name of medicine> \|  \|  \|  \| \| Penicillin \| 250mg/1T \| 3 days \|  \| \|  \| \| \| \|   4. According to the drug prescription, if you took your medicine at 6AM, what time should you take your next dose?  ① 10 AM ② Noon ③ 2 PM ④ 4 PM |
| --- | --- | --- | --- | --- | --- | --- | --- | --- | --- | --- | --- | --- | --- | --- | --- | --- | --- | --- | --- | --- | --- | --- | --- | --- | --- | --- | --- | --- | --- | --- | --- | --- | --- | --- | --- | --- | --- |

※ <The disease pattern diagnosed by health screening at 2005> is shown below.

| 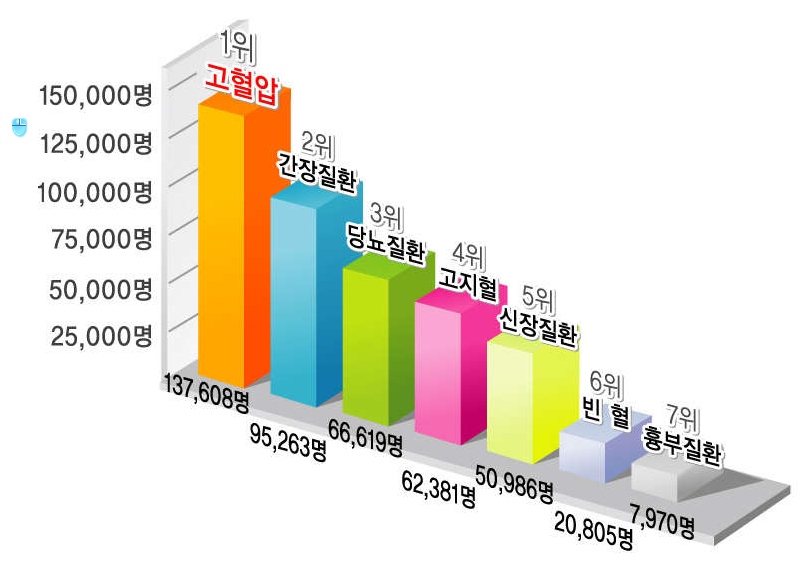  5. What disease is the most highly detected disease through the health screening?  ① Hypertension ② Hepatic disease ③ Diabetes ④ Dyslipidemia |
| --- |

※ Please select the appropriate answer, according to the drug information below.

| [Dosage information]: 10~15㎎/㎏ orally every 4-6 hours as needed,  Maximum daily dose 75㎎/㎏/day. Do not exceed 5 dose in 24 hours. Each dosage according to weight or age is as below.   \| Age \| Weight \| Dosage \| \| --- \| --- \| --- \| \| 4～11 months \| 7.0~9.9㎏ \| 2.5㎖ \| \| 12～23months \| 10.0~11.9㎏ \| 3.5㎖ \| \| 2～3 years \| 12.0~15.9㎏ \| 5㎖ \| \| 4～5 years \| 16.0~20.9㎏ \| 7.5㎖ \| \| 6～8 years \| 21.0~29.9㎏ \| 10㎖ \| \| 9～10 years \| 30.0~37.9㎏ \| 12.5㎖ \|   6. If you are planning to give the medicine to an eight years old child, what is the appropriate dosage?  ① 5㎖ ② 7.5㎖ ③ 10㎖ ④ 12.5㎖ |
| --- | --- | --- | --- | --- | --- | --- | --- | --- | --- | --- | --- | --- | --- | --- | --- | --- | --- | --- | --- | --- | --- |

※ Below is the nutritive component of ramen noodle. Please select the appropriate answer.

| \| Nutritive Facts \| \| \| \| --- \| --- \| --- \| \| Per 1 bag (120g) \| \|  \| \| Amount per bag \| \| % Daily Value* \| \| Calories \| \| 450Kcal \| \| Carbohydrate \| 66g \| 20% \| \| Protein \| 9g \| 15% \| \| Fat \| 18g \| 36% \| \| Sodium \| 1100mg \| 31% \| \| Calcium \| 1600mg \| 23% \| \|  \| \| \| \| *% Daily Value \| \| \|   7. If you have hypertension, you should avoid salty food. In this case which component should you consider the most within the ‘ramen noodle’?  ① calcium ② fat ③ protein ④ sodium |
| --- | --- | --- | --- | --- | --- | --- | --- | --- | --- | --- | --- | --- | --- | --- | --- | --- | --- | --- | --- | --- | --- | --- | --- | --- | --- | --- | --- | --- | --- | --- | --- | --- | --- |
